# Supplementary material for: Acoustic spin rotation in heavy-metal-ferromagnet bilayers
Source: Nat Commun. 2024 Feb 3;15:1013. doi: 10.1038/s41467-024-45317-9 (PMC10837457; doi:10.1038/s41467-024-45317-9)
Supplement: Supplementary file 2 — Supplementary Information [file 41467_2024_45317_MOESM2_ESM.pdf]

Supplementary Materials for

Acoustic Spin Rotation in Heavy-Metal-Ferromagnet Bilayers

Yang Cao,[1] Hao Ding,[1] Yalu Zuo,[1] Xiling Li,[1] Yibing Zhao,[1] Tong Li,[1] Na Lei,[2] Jiangwei Cao,[1] Mingsu Si,[1] Li Xi,[1] Chenglong Jia,[1] Desheng Xue,[1,*] and Dezheng Yang[1,†]

[1]*Key Laboratory for Magnetism and Magnetic Materials of Ministry of Education, Lanzhou University, Lanzhou 730000, China*

[2]*Fert Beijing Institute, MIIT Key Laboratory of Spintronics, School of Integrated Circuit Science and Engineering, Beihang University, Beijing 100191, China*

This PDF file includes:

I. Drift-diffusion model of spin transport

A. Surface-acoustic-wave-induced strain

B. The acoustic magnetization dynamics solved by Landau-Lifshitz-Gilbert equation

C. The derivation of acoustic spin pumping voltage

D. The derivation of acoustic spin rotation voltage

II. Supplementary experimental results

E. The transverse ISHE voltage V_{xy}

F. The Ni thickness dependence of acoustic spin rotation effect

G. Voltage generation from microwave

H. V_{xx} of the sample that inserting a Cu layer between Pt and Ni

[A]uthors to whom correspondence should be addressed:
[*] E-mail: xueds@lzu.edu.cn
[†] E-mail: yangdzh@lzu.edu.cn

I. The H dependence of raw V_{xx} for Pt/Ni

III. References

I. Drift-diffusion model of spin transport

A. Surface-acoustic-wave-induced strain

According to Ref. [1], the longitudinal lattice displacement of SAW can be represented as

$$u_x = \mathrm{Sign}(k) u_{x,0} e^{i(kx-\omega t)}, \tag{S1}$$

with

$$u_{x,0} = u_0\sqrt{1-\xi^2}\left(-e^{k_t z} + \frac{2}{2-\xi^2} e^{k_l z}\right), \tag{S2}$$

where k and $\omega = 2\pi f$ are the wave vector and angular frequency of SAW, respectively. u_0 is the amplitude of SAW. ξ is the ratio of the SAW velocity to the transverse wave velocity, which satisfies the following equation (ν is the Poisson ratio):

$$\xi^6 - 8\xi^4 + 8\xi^2\left(3 - \frac{1-2\nu}{1-\nu}\right) - 16\left(1 - \frac{1-2\nu}{2(1-\nu)}\right) = 0. \tag{S3}$$

k_t, k_l are the transverse wave number and the longitudinal wave number, respectively, and their relationships with k are

$$k_t = |k|\sqrt{1-\xi^2},$$
$$k_l = |k|\frac{(2-\xi^2)^2}{4\sqrt{1-\xi^2}}. \tag{S4}$$

The strain tensor of SAW is defined as

$$\varepsilon_{ij} = \frac{1}{2}\left(\frac{\partial u_i}{\partial x_j} + \frac{\partial u_j}{\partial x_i}\right). \tag{S5}$$

According to Eq. (S1), the longitudinal strain of SAW is

$$\varepsilon_{xx} = i\varepsilon_{xx,0} e^{i(kx-\omega t)}, \tag{S6}$$

with

$$\varepsilon_{xx,0} = |k| u_0\sqrt{1-\xi^2}\left(-e^{k_t z} + \frac{2}{2-\xi^2} e^{k_l z}\right). \tag{S7}$$

For the case that the film thickness is several nanometers, the following relation holds:

$k_l z \ll 1, k_t z \ll 1$, thus

$$\varepsilon_{xx,0} \approx |k| u_0 \sqrt{1-\xi^2} \frac{\xi^2}{2-\xi^2}. \tag{S8}$$

B. The acoustic magnetization dynamics solved by Landau-Lifshitz-Gilbert equation

The Landau-Lifshitz-Gilbert (LLG) equation describing magnetization precession reads [2]

$$\frac{\partial \mathbf{m}}{\partial t} = -\gamma \mathbf{m} \times \mathbf{H}_{\text{eff}} + \alpha \mathbf{m} \times \frac{\partial \mathbf{m}}{\partial t}, \tag{S9}$$

where $\mathbf{m}$ is the unit magnetization of the ferromagnet layer, γ is the gyromagnetic ratio, α is the Gilbert damping constant, and $\mathbf{H}_{\text{eff}}$ is the effective magnetic field acting on $\mathbf{m}$, which is expressed as

$$\mathbf{H}_{\text{eff}} = -\frac{1}{M_{\text{s}}} \frac{\partial E_{\text{tot}}}{\partial \mathbf{m}}, \tag{S10}$$

where M_{s} is the saturation magnetization of the ferromagnet layer and E_{tot} is the total magnetic energy, including Zeeman energy, demagnetization energy and magnetoelastic energy, which is expressed as

$$E_{\text{tot}} = -M_{\text{s}} \mathbf{m} \cdot \mathbf{H} + 2\pi M_{\text{s}}^2 m_z^2 + b \varepsilon_{xx} m_x^2, \tag{S11}$$

where $\mathbf{H} = H(\cos\varphi, \sin\varphi, 0)$ and b is the magnetoelastic coupling coefficient.

Under the influence of SAW, the lattice deforms and causes changes in magnetoelastic energy. $\mathbf{m}$ will precess around its equilibrium position $\mathbf{m}_0 = (\cos\varphi, \sin\varphi, 0)$ under the action of the magnetoelastic effective field. In order to solve the dynamic magnetization conveniently, we make a transformation of the spin frame. We define a new frame *XYZ* in which the *X*-axis points along $\mathbf{m}_0$, as shown in **Fig. S1**.

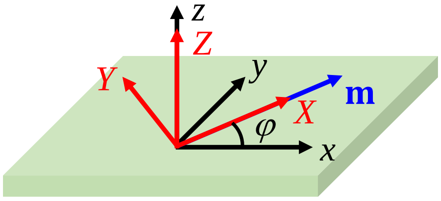

Figure S1. Frame transformation

The transformation matrix that relates the components of $\mathbf{m}$ in the two coordinate systems reads

$$\begin{bmatrix} m_x \\ m_y \\ m_z \end{bmatrix} = \begin{bmatrix} \cos\varphi & -\sin\varphi & 0 \\ \sin\varphi & \cos\varphi & 0 \\ 0 & 0 & 1 \end{bmatrix} \begin{bmatrix} m_X \\ m_Y \\ m_Z \end{bmatrix}. \tag{S12}$$

$\mathbf{m}$ precesses around the X-axis, and we assume that its components in the Y and Z directions are small, i.e., $\mathbf{m} = (1, \delta m_Y, \delta m_Z)$, where $\delta m_Y \ll 1, \delta m_Z \ll 1$. $\mathbf{H}_{\text{eff}}$ is expanded around $\mathbf{m}_0$:

$$\begin{aligned} H_{\text{eff},X} &\approx -\frac{1}{M_s}\left(\frac{\partial E_{\text{tot}}}{\partial m_X}\bigg|_{\mathbf{m}_0} + \frac{\partial^2 E_{\text{tot}}}{\partial m_X \partial m_Y}\bigg|_{\mathbf{m}_0} \delta m_Y + \frac{\partial^2 E_{\text{tot}}}{\partial m_X \partial m_Z}\bigg|_{\mathbf{m}_0} \delta m_Z\right) \\ &\equiv -\frac{1}{M_s}(E_X + E_{XY}\delta m_Y + E_{XZ}\delta m_Z), \\ H_{\text{eff},Y} &\approx -\frac{1}{M_s}\left(\frac{\partial E_{\text{tot}}}{\partial m_Y}\bigg|_{\mathbf{m}_0} + \frac{\partial^2 E_{\text{tot}}}{\partial m_Y^2}\bigg|_{\mathbf{m}_0} \delta m_Y + \frac{\partial^2 E_{\text{tot}}}{\partial m_Y \partial m_Z}\bigg|_{\mathbf{m}_0} \delta m_Z\right) \\ &\equiv h_Y - \frac{1}{M_s}(E_{YY}\delta m_Y + E_{YZ}\delta m_Z), \\ H_{\text{eff},Z} &\approx -\frac{1}{M_s}\left(\frac{\partial E_{\text{tot}}}{\partial m_Z}\bigg|_{\mathbf{m}_0} + \frac{\partial^2 E_{\text{tot}}}{\partial m_Y \partial m_Z}\bigg|_{\mathbf{m}_0} \delta m_Y + \frac{\partial^2 E_{\text{tot}}}{\partial m_Z^2}\bigg|_{\mathbf{m}_0} \delta m_Z\right) \\ &\equiv h_Z - \frac{1}{M_s}(E_{YZ}\delta m_Y + E_{ZZ}\delta m_Z), \end{aligned} \tag{S13}$$

where E_i represents the first-order partial derivative of E_{tot} to m_i, E_{ij} represents the second-order partial derivative of E_{tot} to m_i and m_j, and $h_Y = -E_Y/M_s$ and $h_Z = -E_Z/M_s$ are the Y and Z components of the magnetoelastic effective field induced by SAW, respectively. According to Eq. (S11), we can obtain

$$E_X = -M_s H, E_{ZZ} = 4\pi M_s^2, E_{XY} \approx 0, E_{XZ} \approx 0, E_{YY} \approx 0, E_{YZ} \approx 0, \tag{S14}$$

$$h_Y = \frac{1}{M_s} b\varepsilon_{xx} \sin 2\varphi, h_Z = 0. \tag{S15}$$

Substituting the coordinates of **m** and $\mathbf{H}_{\text{eff}}$ in *XYZ* coordinate system into LLG equation, we obtain

$$\begin{bmatrix} \frac{\partial \delta m_Y}{\partial t} \\ \frac{\partial \delta m_Z}{\partial t} \end{bmatrix} = \frac{\gamma}{M_s} \begin{bmatrix} \delta m_Z E_X - (-M_s h_Z + E_{YZ}\delta m_Y + E_{ZZ}\delta m_Z) \\ -\delta m_Y E_X + (-M_s h_Y + E_{YY}\delta m_Y + E_{YZ}\delta m_Z) \end{bmatrix} + \alpha \begin{bmatrix} -\frac{\partial \delta m_Z}{\partial t} \\ \frac{\partial \delta m_Y}{\partial t} \end{bmatrix}. \tag{S16}$$

With the time-varying strain under SAW, the magnetoelastic effective fields h_Y and h_Z change with time, which leads to the magnetization varying with time. Given the time dependence of the strain (see Eq. (S6)), we assume that $\delta m_Y = m_Y e^{i(kx-\omega t)}, \delta m_Z = m_Z e^{i(kx-\omega t)}$. Thus, Eq. (S16) can be rewritten as

$$\begin{bmatrix} -E_X + E_{YY} - \frac{i\alpha\omega M_s}{\gamma} & E_{YZ} + \frac{i\omega M_s}{\gamma} \\ E_{YZ} - \frac{i\omega M_s}{\gamma} & -E_X + E_{ZZ} - \frac{i\alpha\omega M_s}{\gamma} \end{bmatrix} \begin{bmatrix} \delta m_Y \\ \delta m_Z \end{bmatrix} = M_s \begin{bmatrix} h_Y \\ h_Z \end{bmatrix}. \tag{S17}$$

Substituting Eq. (S14) into Eq. (S17), we obtain

$$\frac{M_s}{\gamma} \begin{bmatrix} \gamma H - i\alpha\omega & i\omega \\ -i\omega & \gamma(H + 4\pi M_s) - i\alpha\omega \end{bmatrix} \begin{bmatrix} \delta m_Y \\ \delta m_Z \end{bmatrix} = M_s \begin{bmatrix} h_Y \\ h_Z \end{bmatrix}. \tag{S18}$$

The solution of Eq. (S18) is:

$$\begin{bmatrix} \delta m_Y \\ \delta m_Z \end{bmatrix} = \frac{\gamma}{(\gamma H - i\alpha\omega)(\gamma(H+4\pi M_s) - i\alpha\omega) - \omega^2} \begin{bmatrix} \gamma(H + 4\pi M_s) - i\alpha\omega & -i\omega \\ i\omega & \gamma H - i\alpha\omega \end{bmatrix} \begin{bmatrix} h_Y \\ h_Z \end{bmatrix}. \tag{S19}$$

We define the resonance linewidth $\Delta H = \frac{\alpha\omega}{\gamma}$ and the resonance field H_r. The relationship between H_r and SAW angular frequency is $\omega = \gamma\sqrt{H_r(H_r + 4\pi M_s)}$. When α^2 is ignored and only the magnetization precession near resonance field H_r is considered (i.e., $H - H_r = \delta$, where δ is a small amount), Eq. (S19) can be approximated as follows:

$$\begin{bmatrix} \delta m_Y \\ \delta m_Z \end{bmatrix} \approx \frac{1}{2H_r + 4\pi M_s} \frac{H - H_r + i\Delta H}{(H - H_r)^2 + \Delta H^2} \begin{bmatrix} H_r + 4\pi M_s & -i\frac{\omega}{\gamma} \\ i\frac{\omega}{\gamma} & H_r \end{bmatrix} \begin{bmatrix} h_Y \\ h_Z \end{bmatrix}. \tag{S20}$$

We define the symmetric lineshape (Lorentz lineshape, *L*)

$$L(H) = \frac{\Delta H^2}{(H-H_r)^2+\Delta H^2} \tag{S21}$$

and the antisymmetric lineshape (Dispersive lineshape, *D*)

$$D(H) = \frac{(H-H_r)\Delta H}{(H-H_r)^2+\Delta H^2}, \tag{S22}$$

then Eq. (S20) becomes

$$\begin{bmatrix} \delta m_Y \\ \delta m_Z \end{bmatrix} = \frac{1}{\Delta H}\frac{D(H)+iL(H)}{2H_r+4\pi M_s}\begin{bmatrix} H_r + 4\pi M_s & -i\frac{\omega}{\gamma} \\ i\frac{\omega}{\gamma} & H_r \end{bmatrix}\begin{bmatrix} h_Y \\ h_Z \end{bmatrix}, \tag{S23}$$

where magnetoelastic effective field $h_Y = \frac{1}{M_\mathrm{S}} b\varepsilon_{xx}\sin 2\varphi, h_Z = 0$. Thus,

$$\begin{bmatrix} \delta m_Y \\ \delta m_Z \end{bmatrix} = \frac{b\varepsilon_{xx}\sin 2\varphi}{M_\mathrm{S}\Delta H}\frac{D(H)+iL(H)}{2H_r+4\pi M_s}\begin{bmatrix} H_r + 4\pi M_s \\ i\frac{\omega}{\gamma} \end{bmatrix}, \tag{S24}$$

where $\varepsilon_{xx} = i\varepsilon_{xx,0}e^{i(kx-\omega t)}$, therefore,

$$\begin{bmatrix} m_Y \\ m_Z \end{bmatrix} = \frac{b\varepsilon_{xx,0}\sin 2\varphi}{M_\mathrm{S}\Delta H}\frac{iD(H)-L(H)}{2H_r+4\pi M_s}\begin{bmatrix} H_r + 4\pi M_s \\ i\frac{\omega}{\gamma} \end{bmatrix}. \tag{S25}$$

C. The derivation of acoustic spin pumping voltage

In the heavy metal (HM)/ferromagnet (FM) system, the spin current $\mathbf{J}_\mathrm{s}^{\mathrm{pump}}$ generated by the acoustic spin pumping (ASP) [3] effect at the HM/FM interface can be expressed as [4]

$$\mathbf{J}_\mathrm{s}^{\mathrm{pump}} = \frac{\hbar}{4\pi} g_{\uparrow\downarrow}\left(\mathbf{m} \times \frac{\mathrm{d}\mathbf{m}}{\mathrm{d}t}\right), \tag{S26}$$

where $\hbar$ is the reduced Planck constant and $g_{\uparrow\downarrow}$ is the spin mixed conductivity that describes the efficiency of the spin passing through the HM/FM interface. It can be seen from Eq. (S26) that the pure spin current generated by ASP effect contains both ac component and dc component. Considering $\mathbf{m} = \left(1, m_Y e^{i(kx-\omega t)}, m_Z e^{i(kx-\omega t)}\right)$, the dc component can be expressed as

$$\mathbf{J}_\mathrm{s}^0 = \frac{\hbar}{4\pi} g_{\uparrow\downarrow}\omega \mathrm{Im}(m_Y^* m_Z)\mathbf{X}. \tag{S27}$$

Substituting Eq. (S25) into Eq. (S27), we obtain

$$\mathbf{J}_\mathrm{s}^0 = \frac{\hbar}{4\pi} g_{\uparrow\downarrow} \left(\frac{b\varepsilon_{xx,0}\sin 2\varphi}{M_\mathrm{s}\Delta H}\right)^2 \frac{H_r+4\pi M_s}{(2H_r+4\pi M_s)^2} \frac{\omega^2}{\gamma} L(H)\mathbf{X}. \tag{S28}$$

The spin current in the HM layer attenuates exponentially along the *z*-direction due to spin diffusion and spin relaxation. With the boundary conditions $\mathbf{J}_\mathrm{s}(0) = \mathbf{J}_\mathrm{s}^0$ and $\mathbf{J}_\mathrm{s}(d) = 0$, the spin current density at position *z* can be obtained by solving the diffusion equation:

$$\mathbf{J}_\mathrm{s}(z) = \mathbf{J}_\mathrm{s}^0 \frac{\sinh[(d-z)/\lambda_N]}{\sinh(d/\lambda_N)}, \tag{S29}$$

where d and λ_N are the thickness and spin diffusion length of the HM layer, respectively.

Averaging Eq. (S29) along the *z*-direction, we can obtain the average spin current generated by ASP effect in the sample:

$$\mathbf{J}_\mathrm{s}^\mathrm{ASP} = \frac{\int_0^d \mathbf{J}_\mathrm{s}(z)\mathrm{d}z}{d} = \frac{\lambda_N}{d}\tanh\left(\frac{d}{2\lambda_N}\right)\mathbf{J}_\mathrm{s}^0 \equiv K(d)\mathbf{J}_\mathrm{s}^0$$
$$= \frac{\hbar}{4\pi} g_{\uparrow\downarrow} K(d) \left(\frac{b\varepsilon_{xx,0}\sin 2\varphi}{M_\mathrm{s}\Delta H}\right)^2 \frac{H_r+4\pi M_\mathrm{s}}{(2H_r+4\pi M_\mathrm{s})^2} \frac{\omega^2}{\gamma} L(H)\mathbf{X}. \tag{S30}$$

The SAW input power *P* is proportional to $\varepsilon_{xx,0}^2/\omega$ in thin films and can be expressed as $P = c_P\varepsilon_{xx,0}^2/\omega$, where c_P is a constant. Using the above relationship, we get

$$\mathbf{J}_\mathrm{s}^\mathrm{ASP} = \frac{\hbar}{4\pi} g_{\uparrow\downarrow} K(d) \left(\frac{b}{M_\mathrm{s}\Delta H}\right)^2 \frac{P}{c_P} \frac{H_r+4\pi M_\mathrm{s}}{(2H_r+4\pi M_\mathrm{s})^2} \frac{\omega^3}{\gamma} L(H)\sin^2 2\varphi\mathbf{X} \equiv J_s\mathbf{X}. \tag{S31}$$

where J_s is the magnitude of the ASP-generated spin current. From Eq. (31), we can see that the spin $\boldsymbol{\sigma}$ of the ASP-generated spin current $\mathbf{J}_\mathrm{s}^\mathrm{ASP}$ is along the $\mathbf{X}$ direction, i.e., the equilibrium magnetization direction.

The spin current in the HM layer is converted into a charge current via the inverse spin Hall effect (ISHE) [5], which reads

$$\mathbf{J}_\mathrm{c} = \theta_\mathrm{SH}\frac{2e}{\hbar}\mathbf{z}\times\mathbf{J}_\mathrm{s}^\mathrm{ASP} = \frac{2\theta_\mathrm{SH}e}{\hbar}J_s\mathbf{Y}, \tag{S32}$$

where θ_{SH} is the spin Hall angle of the HM layer and e is the elementary charge. Therefore, the symmetric ASP voltage in the x-direction is

$$V_{xx}^{S}=L_N E_{\mathrm{ASP}}=\frac{L_N J_{cx}}{\sigma_N}=\frac{L_N \mathbf{J}_{\mathrm{c}}\cdot\mathbf{x}}{\sigma_N}=-\frac{2\theta_{\mathrm{SH}} e L_N}{\hbar\sigma_N}J_s\sin\varphi$$

$$=-\frac{\theta_{\mathrm{SH}} e L_N}{2\pi\sigma_N}g_{\uparrow\downarrow}K(d)\left(\frac{b}{M_{\mathrm{S}}\Delta H}\right)^2\frac{P}{c_P}\frac{H_r+4\pi M_{\mathrm{S}}}{(2H_r+4\pi M_{\mathrm{S}})^2}\frac{\omega^3}{\gamma}L(H)\sin\varphi\sin^2 2\varphi$$

$$\equiv c_{\mathrm{ASP}}L(H)\sin\varphi\sin^2 2\varphi, \tag{S33}$$

where L_N and σ_N are the length and conductivity of the HM layer, respectively, and we have defined $c_{\mathrm{ASP}}=-\frac{\theta_{\mathrm{SH}} e L_N}{2\pi\sigma_N}g_{\uparrow\downarrow}K(d)\left(\frac{b}{M_{\mathrm{S}}\Delta H}\right)^2\frac{P}{c_P}\frac{H_r+4\pi M_{\mathrm{S}}}{(2H_r+4\pi M_{\mathrm{S}})^2}\frac{\omega^3}{\gamma}$.

When H becomes a negative magnetic field, the spin direction $\boldsymbol{\sigma}$ of the injected spin current is changed, thus inducing a sign change of ASP voltage due to ISHE as follows:

$$V_{xx}^{S}(-H)=-c_{\mathrm{ASP}}L(H)\sin\varphi\sin^2 2\varphi. \tag{S34}$$

Eqs. (S33) and (S34) can be written together as

$$V_{xx}^{S}=c_{\mathrm{ASP}}\mathrm{Sign}(H)L(H)\sin\varphi\sin^2 2\varphi. \tag{S35}$$

D. The derivation of acoustic spin rotation voltage

To explain the experimental results, we propose the acoustic spin rotation (ASR) effect via the interface spin orbit interaction. Since the additional ISHE voltage always has a 90° difference in angular dependency with the ASP voltage for injecting any in-plane spin, this implies the existence of a z-direction spin-orbit field $\mathbf{B}_{\mathrm{SO}}$ due to lattice vibration. The spin $\boldsymbol{\sigma}$ of the ASP-generated spin current J_s can be rotated 90° to $\boldsymbol{\sigma}'$ by $\mathbf{B}_{\mathrm{SO}}$ via the spin precession, which reads

$$\mathbf{J}_{\mathrm{s}}^{\mathrm{ASR}}=\alpha J_s\mathbf{B}_{\mathrm{SO}}\times\boldsymbol{\sigma}, \tag{S36}$$

where J_s is the ASP-generated spin current expressed as Eq. (S31), $\mathbf{J}_{\mathrm{s}}^{\mathrm{ASR}}$ is the ASR-

induced spin current with spin $\boldsymbol{\sigma}' \propto \mathbf{B}_{\mathrm{SO}} \times \boldsymbol{\sigma}$, and α is a coefficient.

$\mathbf{J}_{\mathrm{s}}^{\mathrm{ASR}}$ in Eq. (S36) is converted into a charge current via ISHE, which reads

$$\mathbf{J}_{\mathrm{c}}' = \theta_{\mathrm{SH}} \frac{2e}{\hbar} \mathbf{z} \times \mathbf{J}_{\mathrm{s}}^{\mathrm{ASR}} = -\alpha B_{SO} \frac{2\theta_{\mathrm{SH}} e}{\hbar} J_s \mathbf{X}. \tag{S37}$$

Notice that $\boldsymbol{\sigma} = \mathbf{X}$ here.

Therefore, the antisymmetric ASR voltage in the *x*-direction is

$$V_{xx}^{A} = L_N E_{\mathrm{ASR}} = \frac{L_N J_{cx}'}{\sigma_N} = \frac{L_N \mathbf{J}_{\mathrm{c}}' \cdot \mathbf{x}}{\sigma_N} = -\alpha B_{SO} \frac{2\theta_{\mathrm{SH}} e L_N}{\hbar \sigma_N} J_s \cos\varphi$$

$$= -\alpha B_{SO} \frac{\theta_{\mathrm{SH}} e L_N}{2\pi \sigma_N} g_{\uparrow\downarrow} K(d) \left(\frac{b}{M_s \Delta H}\right)^2 \frac{P}{c_P} \frac{H_r + 4\pi M_s}{(2H_r + 4\pi M_s)^2} \frac{\omega^3}{\gamma} L(H) \cos\varphi \sin^2 2\varphi,$$

$$\equiv c_{\mathrm{ASR}} L(H) \cos\varphi \sin^2 2\varphi, \tag{S38}$$

where we define $c_{\mathrm{ASR}} = -\alpha B_{SO} \frac{\theta_{\mathrm{SH}} e L_N}{2\pi \sigma_N} g_{\uparrow\downarrow} K(d) \left(\frac{b}{M_s \Delta H}\right)^2 \frac{P}{c_P} \frac{H_r + 4\pi M_s}{(2H_r + 4\pi M_s)^2} \frac{\omega^3}{\gamma}$.

When H becomes a negative magnetic field, the spin direction $\boldsymbol{\sigma}'$ is changed, thus inducing a sign change of ASR voltage due to ISHE as follows:

$$V_{xx}^{A}(-H) = -c_{\mathrm{ASR}} L(H) \cos\varphi \sin^2 2\varphi. \tag{S39}$$

Eqs. (S38) and (S39) can be written together as

$$V_{xx}^{A} = c_{\mathrm{ASR}} \mathrm{Sign}(H) L(H) \cos\varphi \sin^2 2\varphi. \tag{S40}$$

II. Supplementary experimental results

E. The transverse ISHE voltage V_{xy}

Figure S2 shows the representative optical image of the Hall device.

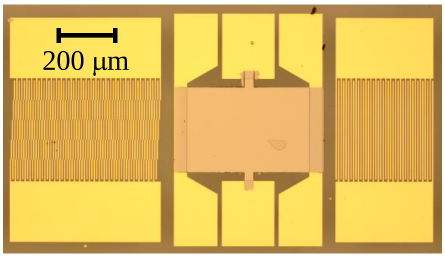

Figure S2. Representative optical microscopy image of the device. A Hall bar made of the HM/FM bilayer is placed between the two interdigital transducers.

We also measured the transverse ISHE voltage V_{xy} of the Hall bar device, which can further prove the acoustic spin rotation model. **Figures S3(a)** and **(b)** show the measured longitudinal ISHE voltage V_{xx} and transverse ISHE voltage V_{xy} of Pt/Ni bilayers, respectively. For the convenience of comparing V_{xx} and V_{xy}, we normalize them both to [-1, 1]. Similar with the discussion of the manuscript, we decompose the asymmetric V_{xx} into two components that are symmetric V_{xx}^{S} [**Fig. S3(c)**] and antisymmetric V_{xx}^{A} [**Fig. S3(e)**] with respect to $\varphi = 90^{\mathrm{o}}$. The angular dependence of V_{xx}^{S} and V_{xx}^{A} can be fitted very well by following functions

$$V_{xx}^{\mathrm{ASP}} = V_{\mathrm{ASP}}\sin\varphi\sin^{2}2\varphi, \quad \text{(S41)}$$

$$V_{xx}^{\mathrm{ASR}} = V_{\mathrm{ASR}}\cos\varphi\sin^{2}2\varphi, \quad \text{(S42)}$$

where V_{ASP} and V_{ASR} represent the amplitudes of acoustic spin pumping voltage induced by $\boldsymbol{\sigma}$ [3] and acoustic spin rotation voltage induced by $\boldsymbol{\sigma}'$, respectively. Since acoustic ferromagnetic resonance exhibit $\sin^{2}2\varphi$ angular dependence [**Fig. 2(d)**], the amplitude of injected spin current also has $\sin^{2}2\varphi$ angular dependence, and this enters $V_{xx}^{\mathrm{ASP}}, V_{xx}^{\mathrm{ASR}} \propto \sin^{2}2\varphi$. The rest contribution of the $\sin\varphi$ ($\cos\varphi$) dependence is due to the projection of $\boldsymbol{\sigma}$ ($\boldsymbol{\sigma}'$) induced ISHE voltage along the *x*-direction, as shown in **Fig. S4**.

When considering the projection of $\boldsymbol{\sigma}$ and $\boldsymbol{\sigma}'$ induced ISHE voltage along the *y*-direction, we can directly write

$$V_{xy}^{\mathrm{ASP}} = -V_{\mathrm{ASP}}\cos\varphi\sin^{2}2\varphi, \quad \text{(S43)}$$

$$V_{xy}^{\mathrm{ASR}} = V_{\mathrm{ASR}}\sin\varphi\sin^{2}2\varphi. \quad \text{(S44)}$$

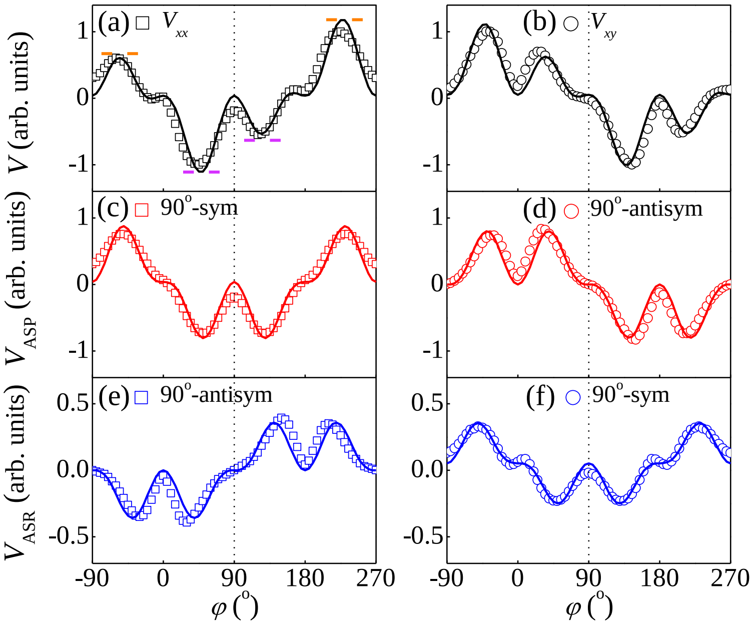

Figure S3. The angular dependence of (a) longitudinal ISHE voltage V_{xx} and (b) transverse ISHE voltage V_{xy} for Pt/Ni bilayers. V_{xx} is extracted into (c) the symmetric and (e) the antisymmetric components with respect to $\varphi = 90^{\circ}$. V_{xy} is extracted into (d) the antisymmetric and (f) the symmetric components with respect to $\varphi = 90^{\circ}$.

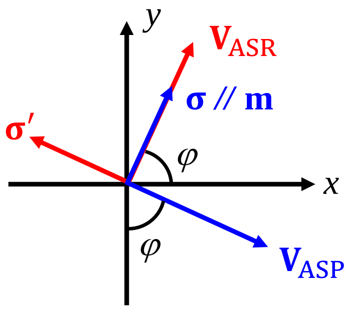

Figure S4. Schematic diagram of $\boldsymbol{\sigma}$ ($\boldsymbol{\sigma}'$) induced ISHE voltage $\mathbf{V}_{\mathrm{ASP}}$ ($\mathbf{V}_{\mathrm{ASR}}$).

According to Eqs. (S43) and (S44), we also decompose V_{xy} into two components that are antisymmetric [**Fig. S3(d)**] and symmetric [**Fig. S3(f)**] with respect to $\varphi = 90^{\circ}$, which can be fitted very well by Eqs. (S43) and (S44), respectively. It should be noted that there is a negative sign difference between V_{xy}^{ASP} in **Fig. S3(d)** and V_{xx}^{ASR}

in **Fig. S3(e)**. This can be easily understood by the following analysis. As shown in **Fig. S4**, when $\mathbf{V}_{\text{ASP}}$ is rotated 90° to $\mathbf{V}_{\text{ASR}}$, the projection along the *x*-direction changes from $\sin\varphi$ to $\cos\varphi$, and the projection along the *y*-direction changes from $-\cos\varphi$ to $\sin\varphi$.

Thus, the angular dependence of the measured Hall voltage is completely consistent with our acoustic spin rotation model.

F. The Ni thickness dependence of acoustic spin rotation effect

We measured V_{xx} of Pt/Ni bilayers with different Ni thicknesses at a fixed SAW frequency *f* = 2.1 GHz. In order to clearly show the acoustic spin rotation effect, we decompose V_{xx} of different Ni thicknesses into symmetric component V_{xx}^{S} and antisymmetric component V_{xx}^{A}. For the convenience of comparing the acoustic spin rotation efficiency, we also normalize V_{xx}^{S} of different Ni thicknesses. V_{xx}^{S} for different Ni thicknesses still satisfy the $\sin\varphi\sin^2 2\varphi$ angular dependences [**Fig. S5(a)**]. While V_{xx}^{A} for different Ni thicknesses all satisfy the $\cos\varphi\sin^2 2\varphi$ angular dependences [**Fig. S5(b)**]. However, we note that the antisymmetric acoustic spin rotation voltage increases with increasing Ni thickness. **Figure S5(c)** shows the acoustic spin rotation efficiency $\eta = V_{\text{ASR}}/V_{\text{ASP}}$ as a function of Ni thickness. As Ni thickness increases, η increases sharply at beginning, and then tends to saturation after 30 nm. We find that these data can be described by the drift-diffusion model $\eta(d) = \eta(\infty)\left(1 - \text{sech}(d/\lambda_{sd})\right)$ [6], where λ_{sd} is the spin diffusion length. By fitting, $\lambda_{sd} = 13.5$ nm, suggesting the range of acoustic spin rotation in ferromagnetic layer.

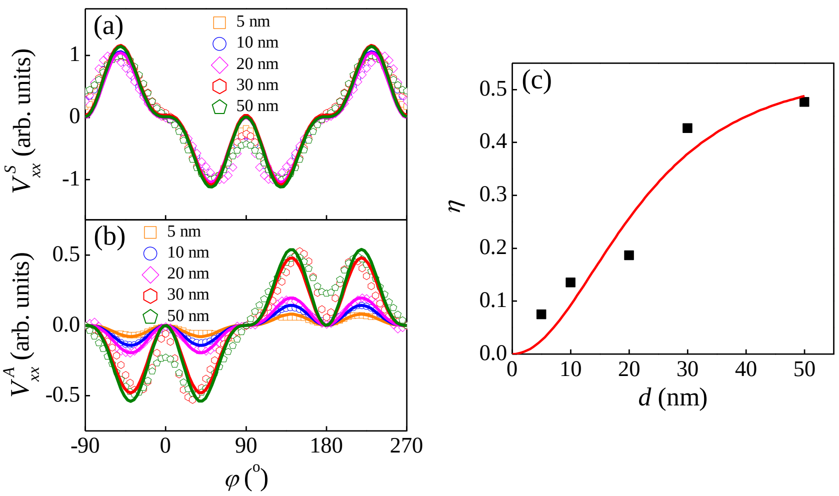

Figure S5. (a) The symmetric and (b) the antisymmetric components of V_{xx} of Pt/Ni bilayers with different Ni thicknesses. (c) Ni thickness dependence of acoustic spin rotation efficiency η.

G. Voltage generation from microwave

The microwave-induced rectification voltage may be mixed in our signal. We can exclude it from the following three aspects.

Firstly, the rectification voltage cannot cause such an angular dependence. When the SAW passes through the Ni stripe, the dynamic strains cause Ni stripe deformation, which further drives magnetization oscillation due to the magnetoelastic coupling. Since the resistivity of the Ni stripe depends on the direction of the magnetization, the SAW-induced magnetization oscillation will induce the variation of the resistivity of Ni stripe. When the time-dependent resistivity is coupled to microwave current, a dc rectified voltage will be generated. According to the work of Chen *et al.* [7], the angular dependence of the SAW-driven rectification Hall voltage in Ni monolayer is $\sin 2\varphi \sin\varphi$ or $\sin 2\varphi$. While the Hall voltage in our work exhibits the $\cos\varphi \sin^2 2\varphi$ and $\sin\varphi \sin^2 2\varphi$ angular dependence.

Secondly, following the reviewer's suggestion, we design a control experiment by

inserting a SiO_2 layer into Pt/Ni bilayer. **Figure S6** shows the longitudinal ISHE voltage V_{xx} of Pt(2)/Ni(30) and Pt(2)/SiO_2(50)/Ni(30) samples. When SiO_2 is inserted between Pt and Ni, V_{ASP} vanishes, demonstrating that the pumping spin current is blocked by the nonmagnetic insulating SiO_2 layer. However, we can note that V_{ASR} also vanishes, suggesting it originates from the pumping spin current, rather than the microwave induced rectification voltage.

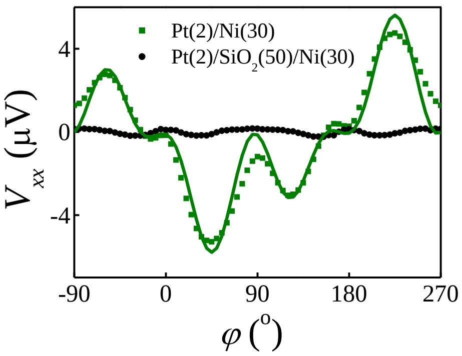

Figure S6. The angular dependence of V_{xx} for Pt(2)/Ni(30) and Pt(2)/SiO_2(50)/Ni(30) samples.

Thirdly, we have carefully filtered out the influence of microwaves in our SAW devices, by optimizing the design to match the impedance. **Figure S7** shows the time-domain results of our SAW device measured by time-resolved methods [8]. The red line in **Fig. S7** is the pulse signal input to interdigital transducer 1, while the blue line is the output signal on interdigital transducer 2. Because the traveling speed of SAW is only 3800 m/s, which is much lower than 3×10^8 m/s of the electromagnetic wave, one can find that SAW-induced signal has a strong delay, but electromagnetic wave-induced signal almost occurs at the same position as the input signal (in the red dotted frame). The almost clean signal in the red dotted frame suggests the influence of microwaves is negligible in our SAW devices.

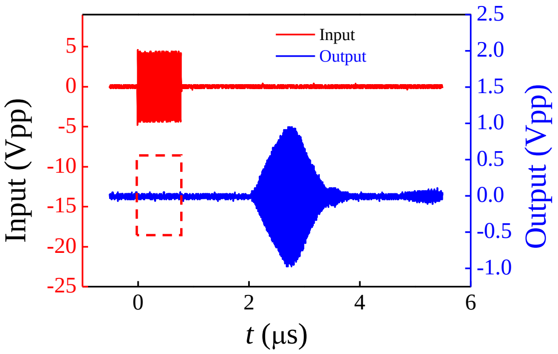

Figure S7. The time-domain measurement results of the SAW device. The input signal (red line) was applied on interdigital transducer 1; the response signal (blue line) was detected on interdigital transducer 2.

H. V_{xx} of the sample that inserting a Cu layer between Pt and Ni

In our acoustic spin rotation model, the acoustic spin rotation effect originates from the Pt/Ni interface. To prove this, we inserted a Cu layer between Pt and Ni. **Figure S8(a)** shows the longitudinal ISHE voltage V_{xx} of Pt(2)/Ni(30), Pt(2)/Cu(6)/Ni(30), and Pt(2)/Cu(50)/Ni(30) samples. When inserting 6 nm Cu between Pt and Ni, the symmetric V_{ASP} was reduced by approximately 40% due to the diffusion of spin current in Cu [see **Fig. S8(b)**]. In contrast, the antisymmetric V_{ASR} decreases more (70%) [**see Fig. S8(c)**], resulting in a 50% reduction of acoustic spin rotation efficiency η. This indicates that the acoustic spin rotation effect originates from the Pt/Ni interface, because the Cu insertion layer destroys the interface between Pt and Ni, leading to a decrease of $\mathbf{B}_{\mathrm{SO}}$. When a thicker Cu (50 nm) is inserted, the spin current vanishes due to the spin relaxation in Cu. As a result, the acoustic spin pumping voltage and acoustic spin rotation voltage both vanish, as shown as the blue scatters in **Fig. S8(a)**.

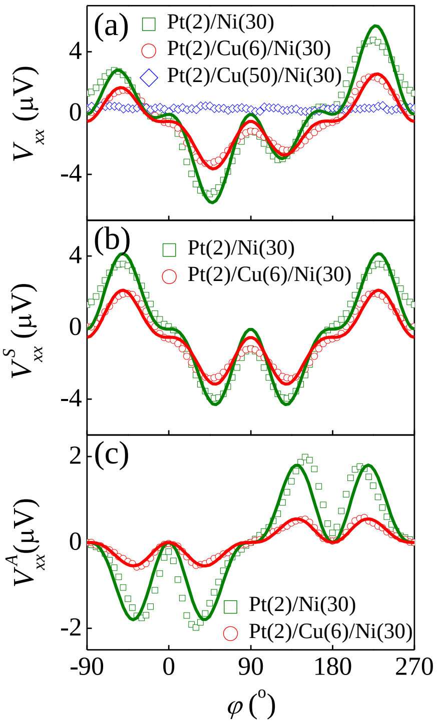

Figure S8. (a) The angular dependence of V_{xx} for Pt(2)/Ni(30), Pt(2)/Cu(6)/Ni(30), and Pt(2)/Cu(50)/Ni(30) samples. We extract V_{xx} into (b) the symmetric and (c) the symmetric components with respect to $\varphi = 90^\mathrm{o}$.

I. The *H* dependence of raw V_{xx} for Pt/Ni

We also measured the raw V_{xx} for Pt/Ni as a function of *H*, as shown in **Fig. S9(a)**. Because acoustic ferromagnetic resonance is strongest near $\varphi = k\pi/2 + \pi/4$ ($k \in$ integer) [**Fig. 2(d)**], we only display raw V_{xx} at -45°, 45°, 135° and 225°. We decompose raw V_{xx} into symmetric [V_S in **Fig. S9(b)**] and antisymmetric [V_A in **Fig. S9(c)**] Lorentz lineshape, according to the method in Ref. [7]. One can find that the antisymmetric signals for all angles are negligible compared to the symmetric signals. This suggests the microwave-induced rectification voltage is negligible in our system [9]. Therefore, the raw V_{xx} is only a symmetric Lorentz lineshape. Besides, we

can observe a significant asymmetry of V_S with respect to $\varphi = 90^{\text{o}}$. V_S is larger at 45° (225°) than that at 135° (-45°). We attribute the asymmetry to the acoustic spin rotation.

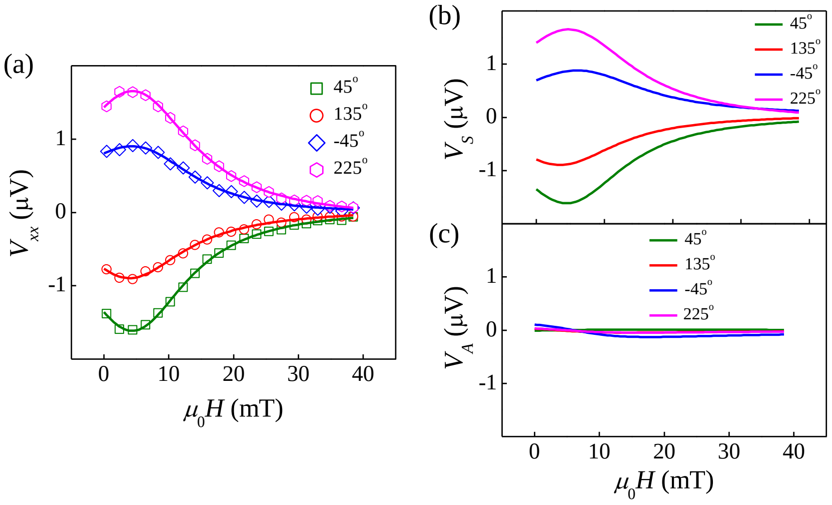

Figure S9. (a) Raw V_{xx} as a function of H at $\varphi = -45^{\text{o}}, 45^{\text{o}}, 135^{\text{o}}$ and 225°. (b) Symmetric (V_S) and (c) antisymmetric (V_A) signals of V_{xx}.

III. References:

[1] T. Kawada, M. Kawaguchi, T. Funato, H. Kohno, and M. Hayashi, *Acoustic Spin Hall Effect in Strong Spin-Orbit Metals*, Sci. Adv. **7**, eabd9697 (2021).

[2] T. L. Gilbert, *Classics in Magnetics A Phenomenological Theory of Damping in Ferromagnetic Materials*, IEEE Trans. Magn. **40**, 3443 (2004).

[3] M. Weiler, H. Huebl, F. S. Goerg, F. D. Czeschka, R. Gross, and S. T. B. Goennenwein, *Spin Pumping with Coherent Elastic Waves*, Phys. Rev. Lett. **108**, 176601 (2012).

[4] D. Wei, M. Obstbaum, M. Ribow, C. H. Back, and G. Woltersdorf, *Spin Hall Voltages from a.c. and d.c. Spin Currents*, Nat. Commun. **5**, 3768 (2014).

[5] E. Saitoh, M. Ueda, H. Miyajima, and G. Tatara, *Conversion of Spin Current into Charge Current at Room Temperature: Inverse Spin-Hall Effect*, Appl. Phys. Lett. **88**, 182509 (2006).

[6] M. Dc et al., *Observation of Anti-Damping Spin–Orbit Torques Generated by in-Plane and out-of-Plane Spin Polarizations in MnPd3*, Nat. Mater. **22**, 591 (2023).

[7] C. Chen, L. Han, P. Liu, Y. Zhang, S. Liang, Y. Zhou, W. Zhu, S. Fu, F. Pan, and C. Song, *Direct-Current Electrical Detection of Surface-Acoustic-Wave-Driven Ferromagnetic Resonance*, Adv. Mater. **35**, 2302454 (2023).

[8] Y. Cao, X. N. Bian, Z. Yan, L. Xi, N. Lei, L. Qiao, M. S. Si, J. W. Cao, D. Z. Yang, and D. S. Xue, *Surface Acoustic Wave-Assisted Spin–Orbit Torque Switching of the Pt/Co/Ta*

Heterostructure, Appl. Phys. Lett. **119**, 012401 (2021).

[9] M. Harder, Y. Gui, and C.-M. Hu, *Electrical Detection of Magnetization Dynamics via Spin Rectification Effects*, Phys. Rep. **661**, 1 (2016).
